# Child health insurance coverage: a survey among temporary and permanent residents in Shanghai

**DOI:** 10.1186/1472-6963-8-238

**Published:** 2008-11-17

**Authors:** Mingshan Lu, Jing Zhang, Jin Ma, Bing Li, Hude Quan

**Affiliations:** 1 Department of Economics, University of Calgary, Calgary, Alberta, Canada; 2 School of Public Health, Shanghai Jiaotong University, Shanghai, PR China; 3 Calgary Health Region, Calgary, Alberta, Canada; 4 Department of Community Health Sciences and Centre for Health and Policy Studies, University of Calgary, Calgary, Alberta, Canada

## Abstract

**Background:**

Under the current healthcare system in China, there is no government-sponsored health insurance program for children. Children from families who move from rural and interior regions to large urban centres without a valid residency permit might be at higher risk of being uninsured due to their low socioeconomic status. We conducted a survey in Shanghai to describe children's health insurance coverage according to their migration status.

**Method:**

Between 2005 and 2006, we conducted an in-person health survey of the adult care-givers of children aged 7 and under, residing in five districts of Shanghai. We compared uninsurance rates between temporary and permanent child residents, and investigated factors associated with child health uninsurance.

**Results:**

Even though cooperative insurance eligibility has been extended to temporary residents of Shanghai, the uninsurance rate was significantly higher among temporary (65.6%) than permanent child residents (21.1%, adjusted odds ratio (OR): 5.85, 95% confidence interval (95% CI): 4.62–7.41). For both groups, family income was associated with having child health insurance; children in lower income families were more likely to be uninsured (OR: 1.96, 95% CI: 1.40–2.96).

**Conclusion:**

Children must rely on their parents to make the insurance purchase decision, which is constrained by their income and the perceived benefits of the insurance program. Children from migrant families are at even higher risk for uninsurance due to their lower socioeconomic status. Government initiatives specifically targeting temporary residents and providing health insurance benefits for their children are urgently needed.

## Background

In China, the 'hukou' system, a system of residency permits dating back to the 1950s which identified a person as an area resident according to the household registration record, formerly imposed strict controls on migration. The 'hukou' system effectively minimized a person's mobility by tying various opportunities and social benefits (including employment, housing, healthcare, and children's education) with her/his official place of residence [1]. Starting in the 1980s, China went through major market reforms and fast economic growth. Such growth was largely unbalanced, not only between urban and rural areas, but also among different regions. On the one hand, reforms in urban areas, including development of a contract labour system, created a large demand for rural migrants. On the other hand, agricultural market reforms produced a large surplus labour supply. More importantly, the emerging private markets for housing and employment opened new opportunities for social mobility [2]. As a result, it created a huge new "floating population", i.e., people who migrated from rural to urban areas, from underdeveloped to developed regions, and from central and western to eastern coastal areas. In 2003, China's floating population reached 140 million, accounting for 10 per cent of the total population and about 30 per cent of the rural labour force [3].

Unlike permanent residents, these migrants and their children (i.e., temporary residents) do not have residency permits, and are not eligible for various social benefits that are tied to residency, for example, their children can only attend designated schools. Thus, their social and economic status is similar to those of illegal immigrants [4]. Furthermore, temporary residents may be at increased risk of poor health because of the physical and psychological stresses involved in migration, resettlement and adaptation to a new community and a new life. Their health needs may not be met due to financial barriers in accessing the healthcare system. The consequences of these problems may be particularly pronounced for children.

Concurrent with its economic upheaval, China's healthcare system went through a series of reforms, changing from the previous centrally-planned and universal healthcare system to a heavily market-based one [5]. Under the new system, there are strong economic incentives for healthcare providers to engage in competition, waiting lists have been largely eliminated, and for those who can afford to pay, the quality and choice of care have improved significantly. During this period, China's national health expenditures increased from 110.2 hundred million Chinese RMB (renminbi) in 1978 to 7590.3 hundred million Chinese RMB in 2004 [6]. Despite such a dramatic increase in healthcare costs, children are no longer covered under public insurance.

In the urban areas, prior to healthcare reform, two types of public health insurance programs (Labor Health Insurance and Government Health Insurance programs) covered the majority of residents, including dependent children. After the reform, these programs were replaced by a new urban employee health insurance program. This new public insurance program provides insurance coverage to all enterprise employees only; their dependent children are no longer covered [6]. In the rural areas, all residents were previously covered by the Cooperative Medical Scheme (CMS), a form of collective economy prepaid health security program. After the reform, the old CMS literally collapsed. Rural healthcare, for both adults and children, has reverted primarily to private financing (self-pay) [7]. In recent years, the Chinese government has been implementing various forms of new CMS pilot projects all over the country, aiming to re-establish CMS in the rural areas and increase insurance coverage among the rural population [8,9]. This new CMS provides limited coverage to families who enrol, including their children. In addition, starting in the early 1980s, private (or commercial) health insurance plans, offered by life insurance companies, were introduced in both urban and rural areas. The development of the private health insurance market in China is still at a very preliminary stage. According to the Third National Health Survey in China, only 5.6 per cent of the population was covered by commercial health insurance [10].

In summary, under the current healthcare system, children in both urban and rural areas, particularly those of migrant families, have become one of the most vulnerable populations in China in terms of healthcare access. Uninsurance among children has long been attracting attention from health policy makers and researchers in developed countries such as the US [11]. Existing literature has indicated that children with insurance are more likely to have a usual source of care [12-14], to have access to preventive care [15,16], to get the healthcare services they need [17], to have improved social and emotional development [18], and to be better equipped to do well in school, which leads to higher productivity in the future [19,20]. As a result, the State Children's Health Insurance Program (SCHIP) was designed and implemented largely to expand health insurance coverage for children in the US [21,22]. Furthermore, immigrants in the US have been found to more likely to be uninsured than U.S.-born populations and have been identified as a vulnerable population [23-26].

Reducing disparities and better protecting vulnerable populations has become one of the Chinese government's biggest challenges. Recognizing the magnitude and consequences of low insurance coverage for children, the government is seeking solutions in not-for-profit cooperative insurance programs. Shanghai is one of the few cities in China that offer such a program. Shanghai Children Hospital Care Aid (SCHCA) was established in 1999 under the joint efforts of the Shanghai Red Cross, Shanghai Education Commission, and Shanghai Bureau of Health, and was intended to offer hospital coverage for children at a low premium. The SCHCA premium is currently set at 60 Chinese RMB (about 8 US dollars) per year for age 0–5; 50 Chinese RMB per year for age 6–18. (As a reference point, the average annual household income in Shanghai in 2006 was 20,668 Chinese RMB [27]) In China, one has to pay a significant deposit at admission in order to be hospitalized. Enrolees of SCHCA, when hospitalized in designated hospitals, only pay 50 per cent of this deposit. Upon discharge, 50 per cent of the total medical expense above the deductible is also covered (depending on the type of hospital, the deductible ranges from 100 to 300 Chinese RMB). In addition, SCHCA covers 50 per cent of the fees for specialist outpatient visits after hospital discharge for leukemia, hemophilia, aplastic anemia, and cancer, as well as the cost of renal dialysis prior to transplant, and the cost of anti-rejection drugs after the surgery. SCHCA was initially offered only to permanent child residents in Shanghai. As of September 1^st ^2004, children who were born between September 1^st^, 1998 and August 7^th^, 2003 to temporary residents are also eligible to enrol [28].

Do disparities exist between permanent and temporary residents in terms of child health insurance coverage? How effective is SCHCA in terms of expanding child health insurance coverage among both permanent and temporary residents in Shanghai? What are the determinants of families' decisions to purchase child health insurance? In order to shed light on these important questions, we conducted an in-person survey of the parents or guardians of temporary and permanent child residents (age 7 or younger) in five districts in Shanghai. Using this unique child health survey data, we described and compared uninsurance rates of temporary and permanent child residents in Shanghai, where both public and private insurance are available. We investigated factors associated with child health uninsurance and identified subgroups of temporary child residents who are at high risk of uninsurance.

## Method

### Study population: The Shanghai Child Residents Health Survey

We conducted a survey of children aged 7 or younger from 2005 to 2006 in Shanghai. Five out of the 19 districts in Metropolitan Shanghai were included in the survey: Baoshan, Hongkou, Zhabei, Minhang and Changning. These districts were selected because they have the highest densities of temporary residents in Shanghai. Although these districts are not randomly selected from all districts in the Shanghai metropolitan area, they are representative in the following way: Changning is a high income inner-city district; Hongkou and Zhabei are lower income inner-city districts; Baoshan and Minhang are suburban districts with a large number of temporary residents.

The sample in this survey was randomly selected from children going for regular checkups at community child health clinics and those attending day care centres. (It is far less feasible to reach families, especially temporary residents, who do not bring their children for checkups or send their children to day care centres.) Within each district, both temporary and permanent child residents in the child health clinics and day care centres were clustered by seven age groups (age 0–1, 1–2, ...., 6–7) and by sex. Children who were younger than three were recruited from community child health clinics; those who were older than three were recruited from day care centres. Within each cluster, a sample of 15 children was collected. Participants were randomly selected as people arrived at the child health clinics or day care centres; parents or guardians were surveyed until the quota of 15 children was reached for each cluster. This resulted in about 2,000 households in our sample.

The survey was conducted by teams in each district consisting of two to three pediatricians and children's healthcare nurses who work in the community child health clinics. Before the survey, all interviewers were trained by the principal investigator (Jing Zhang) who designed and tested this survey. Interviewers visited child health clinics and day care centers to conduct the survey. Ethics approval for this study was obtained from Shanghai Jiaotong University. Interviewers explained the purposes and confidentiality of the survey, and then invited parents or guardians of the children to participate in the survey. Respondents could choose not to participate; their participation in the survey was accepted as oral consent. The survey response rate was over 90 per cent. The completeness of questionnaires was checked by a district survey manager at the end of every day. If there was missing information on the survey, individuals would be telephoned and resurveyed if possible. The questionnaires were then submitted to and reviewed by the principal investigator.

### Dependent Variable: Child Health Insurance

Children in Shanghai may hold three types of health insurance: SCHCA, CMS, or private insurance. As described earlier, there is no universal public insurance for children in China. SCHCA is one of the first few cooperative insurance programs offered to children. It was offered initially to permanent child residents only and subsequently extended to all child residents in Shanghai. For migrant residents coming from rural areas, a small number of families hold some form of CMS, which provides insurance coverage to both adults and children. Other than these two forms of cooperative public insurance, the third insurance option for children is private insurance.

### Independent Variables

There is a large literature in health economics studying what are the factors affecting people's decisions of purchasing health insurance. Individual and household social-economic factors, such as age, sex, education, employment, income and family structure, are found to be important determinants of health insurance [29,30]. The actual or perceived health status of an individual, a predictor of expected healthcare expenditure, is also found to be associated with the decision to purchase health insurance [31-33]. Similarly, expected healthcare expenditure is another predictor of the health insurance purchase decision [34]. How individuals perceive risk is also found to be an important factor [35]. Following the existing literature, we included both individual and household characteristics as independent variables in our model.

#### Child characteristics

The survey collected information on the child's age, sex, and health status. An ordinal variable was created to indicate whether a child's age was 0–1, 1–2, 2–3, ... or 6–7. There are three measures of child health status: 1) the child's overall growth as assessed by the parent (three categories: "good", "fair" and "poor"); 2) whether the child had an illness in the previous two weeks for which a doctor was seen; and 3) whether the child was hospitalized during the past year.

#### Household Characteristics

We include in all our models household monthly income, mother's education, mother's occupation, father's education, father's occupation, family size, and parents' opinion on health insurance price. For monthly household income, respondents were asked to choose from the following six categories: < 500 Chinese RMB a month, 500 to 1,000 RMB, 1,000 to 3,000 RMB, 3,000 to 6,000 RMB, 6,000 to 10,000 RMB, and above 10,000 RMB. We regrouped this variable into three categories: low income (below 3000 Chinese RMB), middle income (between 3000 and 6000 Chinese RMB, and high income (above 6000 Chinese RMB) (average monthly household income in Shanghai was around 3000 Chinese RMB in 2003; 1 Chinese RMB = 0.13 U.S. dollars.) The child's family size included living with parents only, with both parents and grandparents, a single-parent family, or others. Given that the per centage of single-parent families is very low in our sample (less than 1 per cent), we redefined this variable into two categories: child living with parents only or not. To shed light on public opinion about health insurance cost, our survey asked the parents whether they thought health insurance was "too expensive, acceptable, or don't care".

### Statistical Analysis

Descriptive statistics were employed to illustrate the child and parent characteristics of the study population; the characteristics included sociodemographic characteristics, child health status, and family structure. We tested these characteristics for statistical differences between permanent and migrant residents. Frequencies of variables in the survey were not weighted because sampling weights were not available. Finally, a binary logistic model was used to analyze an individual household's choice between having no child health insurance and having child health insurance. Stepwise regression was used to select model predictors.

## Results

### Descriptive results

We excluded 7 duplicate observations and those with missing data, resulting in a valid sample size of 1,907 children. Table 1 presents the characteristics of both children and parents in our study population. Among the 1907 households in our survey, 960 were temporary residents and 947 were permanent residents. The mean age of children in the survey was 3.4 years.

**Table 1:** Characteristics of study population (%)

	**All Residents** **N = 1907**	**Temporary Residents** **N = 960**	**Permanent Residents** **N = 947**
**Children characteristics**			
Male	51.1	51.5	50.8
Mean age (s.d.*^2^)	3.4(1.9)	3.4(1.9)	3.4(1.9)
Self-perceived health status			
Good	52.0	47.7	56.2
Fair	42.3	45.6	38.9
Poor	5.7	6.7	4.9
Illness within 2 weeks	18.3	15.4	21.2
Hospitalization within 1 year	3.1	2.7	3.5
**Parents characteristics**			
Average family income (Yuan/Month)			
Below 3000	40.3	53.1	27.2
3000–6000	42.0	35.0	49.0
Above 6000	17.7	11.9	23.8
Mother's education			
Elementary school	5.4	10.2	0.5
High school	63.3	78.5	47.9
College	31.3	11.3	51.6
Mother's occupation			
Labour	18.9	15.5	22.3
Official	17.5	7.0	28.1
Business	37.6	42.1	33.1
Unemployed	26.1	35.4	16.6
Father's education			
Elementary school	2.1	4.1	0.0
High school	59.0	76.6	41.2
College	38.9	19.3	58.8
Father's occupation			
Labour	27.2	24.9	29.5
Official	20.6	12.5	28.9
Business	49.7	60.6	38.6
Unemployed	2.5	2.0	3.0
Child living with parents only	60.7	66.8	54.6

*Notes:

1. P-values are calculated from two-tailed chi-square test;

2. Standard deviation is reported in the parentheses.

More permanent than temporary child residents were assessed by their parents as having good growth (56.2% versus 47.7%, p < 0.001), were sick during the last two week (21.2% versus 15.4%, p < 0.001), or were hospitalized (3.5% versus 2.7%, p < 0.001). Many more temporary residents fell into the low income category (53.1% versus 27.2%, p < 0.001).

For both parents, the education level of temporary residents was much lower than of permanent residents: 10.2% of the mothers who were temporary residents had only an elementary school education (versus 0.5%, p < 0.001), and only 11.3% attended college and above (versus 51.6%, p < 0.001); similar contrasts existed for the fathers' education. There was also a large difference in mother's and father's occupation. Among temporary residents, both mothers and fathers were less likely than their permanent resident counterparts to be labourers (15.5% versus 22.3% for mothers; 24.9% versus 29.5% for fathers; p < 0.001), officials and professionals (7.0% versus 28.1% for mothers; 12.5% versus 28.9% for fathers; p < 0.001); more likely to be in sales and trade related business (42.1% versus 33.1% for mothers; 60.6% versus 38.6% for fathers; p < 0.001); and more likely to be unemployed (mothers only: 35.4% versus 16.6%; p < 0.001). Temporary child residents were more likely to be living in a three-person only family compared with permanent child residents (66.8% vs. 54.6%, p < 0.001).

### Child health insurance coverage

The overall insurance rate in our sample was 56.5%. The insurance rate was much higher among permanent child residents, reaching 78.9%, compared with the 34.4% insurance rate among temporary child residents (see Table 2). Among the permanent child residents who had health insurance, 56.5% had SCHCA, and 36.8% had private insurance. Among the temporary residents, 22.4% had SCHCA, 0.3% had CMS, and 70.3% had private insurance.

**Table 2:** Insurance coverage and type (%)

	**All Residents**	**Temporary Residents**	**Permanent Residents**	P-value *^3^
Insurance coverage	N = 1907	N = 947	N = 960	< 0.001
	56.5	34.4	78.9	
Insurance type among people insured	N = 1077	N = 330	N = 747	< 0.001
SCHCA*^1^	46.1	22.4	56.5	
CMS*^2^	0.1	0.3	0.0	
Private	47.1	70.3	36.8	
Unknown	6.7	7.0	6.7	

* Notes:

1. Shanghai Children Hospital Care Aid (SCHCA);

2. Cooperative Medical Schedule (CMS);

3. P-values are calculated from two-tailed chi-square test.

Parents' opinions on the cost of health insurance are summarized in Table 3. Among the uninsured population, there is no significant difference between permanent and temporary residents in terms of views on the price of health insurance. Among those who are insured, permanent residents were more likely to find the price of health insurance acceptable than temporary residents. Among both the uninsured and insured populations, there is a significantly higher percentage of temporary residents who answered that they "don't care". This indicates that child health insurance is a lower priority for temporary residents.

**Table 3:** Parent's opinions on price for health insurance (%)

	**All Residents**	**Temporary Residents**	**Permanent Residents**	P-value*
Uninsured population	N = 830	N = 630	N = 200	0.16
Too expensive	21.9	23.0	18.5	
Acceptable	70.7	69.1	76.0	
Don't care	7.4	7.9	5.5	
Insured population	N = 1077	N = 330	N = 747	< 0.001
Too expensive	19.1	24.2	16.9	
Acceptable	76.1	68.5	79.4	
Don't care	4.8	7.3	3.8	
Total population	N = 1907	N = 960	N = 947	< 0.001
Too expensive	20.4	23.4	17.2	
Acceptable	73.7	68.9	78.7	
Don't care	5.9	7.7	4.1	

• Note: P-values are calculated from two-tailed chi-square test.

### Risk adjusted analysis results

Consistent with the descriptive results, after controlling for child and parent characteristics, temporary child residents were significantly more likely to be uninsured compared with permanent child residents (odds ratio: 5.85, 95% confidence interval: 4.62–7.41, See Table 4). The older a child was, the less likely the child was uninsured. Illness within the last two weeks requiring a doctor's visit was significantly associated with having insurance. Children from the lowest income families were less likely to be insured. Lower education in both the mother and the father was found to be significantly associated with child uninsurance. Children living with parents only were more likely to be uninsured than those who live with parents and grandparents. This is likely due to the fact that grandparents bring in an additional source of family income and increase the family's ability to purchase health insurance. Other variables, such as gender, health status measured by growth, mother and father's occupation, or opinion about price of health insurance were not found to be significantly correlated with having child insurance.

**Table 4:** Adjusted odds ratio (95% confidence interval) for uninsured

**Variables**	**OR (95% CI)**
Temporary residents	5.85 (4.62–7.41)
Male	1.03 (0.83–1.28)
Age (every 1 year increase)	0.74(0.69–0.78)
Self-perceived health status	
Good	1.00
Fair	4.04 (0.80–1.26)
Poor	0.89 (0.56–1.43)
Illness within 2 weeks	0.59 (0.44–0.78)
Hospitalization last year	0.61 (0.31–1.16)
Family Income (Yuan/month)	
below 3000	1.96 (1.40–2.96)
3000–6000	1.14 (0.83–1.56)
Over 6000	1.00
Mother's education of Elementary School	2.13(1.12–3.72)
Father's education of Elementary School	2.42 (0.87–6.79)
Mother's Occupation	
Official	1.00
Labour	1.09 (0.70–1.67)
Business	1.22 (0.84–1.77)
Unemployed	1.35 (0.91–1.99)
Father's Occupation	
Official	1.00
Labour	0.82 (0.56–1.19)
Business	0.97 (0.70–1.35)
Unemployed	0.99 (0.47–2.07)
Child living with parents only	1.32 (1.06–1.65)
Opinion on Insurance price	
Acceptable	1.00
Expensive	1.20 (0.91–1.57)
Don't Care	1.33 (0.85–2.10)

We also calculated an adjusted odds ratio for the likelihood of take-up of SCHCA among residents who hold either SCHCA or private insurance. The result indicates that compared with permanent residents, temporary residents are less likely to enrol their children in SCHCA and more likely to buy private insurance (odds ratio: 0.24, 95% confidence interval: 0.17, 0.33). This is consistent with the descriptive results presented in Table 2. As stated earlier, up until 2004, only children who were permanent residents in Shanghai were eligible to enrol in SCHCA. The only option for temporary residents was to purchase private insurance. Since 2004, SCHCA has extended its coverage to children who are temporary residents. However, this information has not been delivered effectively to temporary residents; many temporary residents are still not aware of this benefit.

To test how robust our results are, we used different model specifications (results not presented but available upon request). Interaction terms between being a temporary child resident and variables such as family income, mother's and father's education were included in the models. However, the interaction terms were not statistically significant and the above main results in Table 4 remained robust.

## Discussion

Our study demonstrated that temporary child residents were more likely to be uninsured than permanent child residents in Shanghai. Regardless of their resident status, most of them are not covered under the public health insurance system, making them one of the largest vulnerable populations. Despite the low premium and extended eligibility of SCHCA, the uninsurance rate is still high among both permanent (21.1%) and temporary child residents (65.6%) in our survey sample. This presents a serious concern on the effectiveness of cooperative insurance, such as SCHCA, in eliminating uninsurance among children. One likely reason is SCHCA's high coinsurance rate; even with SCHCA, the child's family still needs to pay a significant part of the total cost. This is consistent with the existing literature on immigrant status and health insurance.

For both temporary and permanent residents, child health status (measured by illness within the last two weeks requiring a doctor's visit), family income, mother and father's education level are found to be significantly associated with the decision to purchase child health insurance: those with better health status, and those in lower income or lower education families are more likely to be uninsured. This is consistent with the existing literature on determinants of health insurance [36-39].

We also found that even with SCHCA eligibility extended to temporary residents in Shanghai, temporary child residents are still significantly more likely to be uninsured compared with permanent child residents. There are several possible barriers to health insurance coverage for temporary residents. The first one is affordability: temporary residents tend to have lower household incomes than permanent residents. The second one is perceived benefit. Temporary residents may be more uncertain about their future need for healthcare in the local healthcare system, and therefore more reluctant to purchase health insurance. The third one is a knowledge issue. With less social support, temporary residents may be less informed about the insurance options available. Lastly, temporary residents tend to be more risk-taking than permanent residents, and thus may be more likely to think their children do not need to have health insurance. In addition, we found that among those who decided to insure their children, temporary residents are much less likely than permanent residents to choose SCHCA and more likely to purchase private insurance.

Our results call for stronger government support of child health insurance programs. The policy implications from our study are three-fold. First, one of the factors responsible for low participation in SCHCA among temporary residents is poor outreach. Campaigns to increase temporary residents' awareness of existing child health insurance programs and their benefits should be launched. Second, government initiatives specifically targeting temporary residents and providing health insurance benefits for their children are urgently needed. Currently, the Chinese government is starting to establish new basic health insurance programs for urban residents including their dependent children. Premiums in these programs are paid jointly by the government, the parents' employer, and the parents. Similar basic insurance programs for temporary residents should also be introduced. Third, there is no law on child health insurance in China. Government legislation is needed to protect children's health.

This study has several limitations. Due to difficulties in reaching temporary residents who are of low socioeconomics status (e.g., construction workers), our survey over-sampled temporary residents who were relatively well educated and had higher incomes. Second, our survey was conducted in the Shanghai metropolitan area, which is one of the most affluent cities and provides one of the best job markets for migrants in China. Only families of children who went to child health clinics for regular checkups and/or those who attended child care centers were sampled. In other words, the actual uninsurance rates and the gap in child insurance between temporary and permanent residents in Shanghai could be much larger than our estimates. Furthermore, these findings could be even more pronounced in other less developed regions in China. In addition, measures of child health status in this study are self-reported and are subject to reporting error. We were not able to assess whether health insurance has improved healthcare access and child health due to lack of data on health services utilization and health outcomes. Our survey did not incorporate instruments to measure and compare quality of care between insured and un-insured children. These limitations in both survey method and content should be addressed in future survey design to allow us to further tackle the questions around health insurance, access, utilization, and outcomes among children in China.

## Conclusion

Investing in children's health coverage is a cost effective way to improve population health and social welfare. Children rely on their parents to make the insurance purchase decision, which is limited by their income and the perceived benefits of the insurance program. Children from migrant families are at even larger risk for uninsurance due to their social and economic status. Our findings will help health policy makers' understanding of the magnitude of health uninsurance in children, and the disparities in coverage between permanent and temporary child residents in China. Our study sheds light on how to reduce such disparities and improve healthcare for vulnerable children.

## Competing interests

The authors declare that they have no competing interests.

## Authors' contributions

ML contributed to the statistical analysis and interpretation; drafted and finalized the manuscript. JZ contributed to the study design, survey conduction and supervision, as well as interpretation. JM contributed to the study design, interpretation and writing of the manuscript. BL contributed to the data management, statistical analysis and interpretation. HQ contributed to the statistical analysis, the interpretation and writing of the manuscript. All authors read and approved the final manuscript.

## Pre-publication history

The pre-publication history for this paper can be accessed here:


